# Treatment of Abscesses in Cavities with Rigid Walls

**Published:** 1890-03-08

**Authors:** 


### TREATMENT OF ABSCESSES IN CAVITIES WITH RIGID WALLS.

Discussing the treatment of these, Professor Küster says that while the early opening of abscesses in general at the most dependent point, and in the case of large accumulations of pus, of making a counter opening, are generally approved by all intelligent surgeons, the application of the same principles to those with rigid walls is too often ignored. Thus it is not uncommon to see abscesses of the antrum opened in the nose, though the aperture is then above the floor of the cavity. He makes a *subperiosteal* incision alongside of the upper jaw, to a great extent avoiding the formation of a permanent fistulous communication between the antrum and the mouth, since the flap of periosteum and mucous membrane rarely fails to unite, and even if it does, it tends to keep the aperture closed.

